# Attitudes and perceptions of teachers toward mental health literacy: A case of Odzi High School, Mutare District, Zimbabwe

**DOI:** 10.3389/fpsyg.2022.1003115

**Published:** 2022-11-10

**Authors:** Theresi Sibanda, Innocent Sifelani, Maurice Kwembeya, Memory Matsikure, Sifikile Songo

**Affiliations:** Department of Psychology, Manicaland State University of Applied Sciences, Mutare, Zimbabwe

**Keywords:** adolescents, attitudes, mental health literacy, perceptions, schools, teachers

## Abstract

In dealing with adolescent mental health, teachers and school institutions have a critical role in minimizing the challenges of mental health. However, the success of such a measure is dependent on teachers’ attitudes and perceptions of mental health literacy. Adolescent mental health disorders have become a major global concern with heightened increases and prevalence in secondary schools. In Zimbabwe, a dearth of knowledge exists in understanding the perceptions and attitudes of teachers in maximizing student mental health and positive educational outcomes. The aim of the research was to understand the attitudes and perceptions of teachers toward mental health literacy. Using a qualitative approach to understanding attitudes and perceptions, a case study was carried out at Odzi High School. A total of 12 teachers were purposively sampled for data generation on knowledge, attitudes, perceptions, and strategies that can be used to inform adolescent mental health. Data were collected using semi-structured interview(s). Emergent themes of the study were knowledge, stigma, training, and development. Using thematic analysis, the data showed that there was generalized knowledge of mental health and there were disparities in effective and efficient responses to gender sensitivity issues. Additionally, this knowledge was not put into practice because of attitudes and perceptions toward mental health. Recommendations from the study include developing of mental health training for teachers, equipping teachers with skills in psychosocial support in schools’ mental health, and enabling teachers to be able to link adolescents to appropriate care within the school and community-based settings. Teachers have the responsibility of promoting the wellbeing of the students, thus minimizing the adverse effects. Outcomes for positive mental health may be the result of teachers’ informed perceptions and positive attitudes on mental health literacy.

## Introduction

Millennium trends on adolescent mental health in schools show that teachers perceptions and attitudes on mental health literacy (MHL) have a major impact on health. Teachers’ traditional role of equipping learners in academic subjects has been outgrown by the need to address the “hidden curriculum”and mental health. Graham et al. (2011) concur that there has been a shift in the role of teachers in students’ lives, as it no longer consists of simply educating students in the academic fields of Math, English, and Science. Emerging needs for students’ psychological and emotional needs transfer to increased responsibilities for teachers to undertake roles to address students’ wellbeing. This is largely due to the proximity of teachers with students, and this has made them *de facto* members of mental health teams (Doll et al., 2014), as well as their act of “loco parenthesis.” Johnson et al. (2011) point out that on a daily basis, teachers interact with more than 100 learners per day. Therefore, teachers have a critical role in having the ability to note behavioral changes and mental health challenges, and needs of learners (Bruland et al., 2017). Teachers further believe that school structures are critical to support learners, and their perception is that there should be a broad common school approach for learners faced with mental health challenges, as well as have good and strong networks to other support services (Schulze et al., 2019). On this account schools provide opportunities for raising awareness, promotion, and prevention of mental health disorders. Given the substantial amount of time that adolescents spend at school combined with the proximity and interactive and loco parental relationship, schools become a fertile environment to provide psychosocial and support activities for adolescent mental health (Kieling et al., 2011; Kutcher et al., 2015). Relatively, directed efforts in addressing mental health issues at schools by teachers may prevent and reduce the severity of depressive symptomology and the development of mental health disorders (Fazel et al., 2014). Improving knowledge and capacity to target groups (teachers) helps them to flag mental disorders at an early stage, correct classification of symptoms, and be able to gather reliable information and direct contacts for assistance. Such measures are likely to increase motivations for health-seeking behaviors and treatment, consequentially leading to better mental health outcomes and improved quality of life in the long term (Seven et al., 2021). Li et al. (2022), consent that those educators have a significant social role in attending to MHL; therefore, their ability in MHL is important to develop those that they educate. This implies that teachers have a critical role to play in addressing mental health disorders. Arguably, when teachers attend to the mental needs and wellbeing of learners, they ensure that adolescents develop to their full potential and achieve positive educational outcomes. However, the challenge is that school authorities fail to recognize risk learners (Johnson et al., 2011). A generalized view is that the concept of health literacy (teachers) has been given little attention, and more specifically, an overview of MHL among young people (secondary school learners) is lacking. Research evidence has shown that there is a link between health literacy and behavior among young people, which indicates a poignant need for the promotion of health literacy (Fleary et al., 2018). Identification of risk learners places value on teachers’ attitudes, perceptions, and attitudes on MHL for addressing the wave of increases and prevalence in mental disorders among adolescents.

The onset of mental health disorders has been reported to begin between ages 10 and 14 years, which are crucial stages of social, emotional, and cognitive development. The onset of mental health disorders has long-term effects on developmental transitions and learning experiences (World Health Organisation [WHO], 2020). Contextually, the global burden of the disease shows that 10–20% are mental health problems affecting children and adolescents (Ontario Ministry of Education, 2013). In sub-Saharan Africa, the burden of common mental health disorders (CMDs) has doubled among young adults and adolescents in the past 30 years (Gouda et al., 2019). In Zimbabwe, the prevalence of common mental disorders among adolescents in rural Zimbabwe was more than 50% in adolescents and young adults who screened positive for CMDs (Brostrom et al., 2021). Consequently, there exists a huge treatment mental health gap among children and adolescents (Kieling et al., 2011; Brostrom et al., 2021) with most mental health disorders being undetected and untreated among children and adolescents (World Health Organisation [WHO], 2014). In general, the prevalence and incidence of mental disorders are rooted in structural social adversity. Challenges like poverty, genetic predispositions, child-headed homes, family dysfunction, bullying pressure of academic work, and domestic violence are in part or whole contribute to adolescent mental health (Chibanda et al., 2016; Reupert and Maybery, 2016). These statistics may be conservative bearing the context of COVID-19 impact on the student’s mental health. Rao and Rao (2021) state that there was an increased vulnerability to mental health, as 81% of students experienced high-stress levels during the COVID-19 pandemic. Return to normalcy coupled with structural challenges points to the dire need for attention to adolescent mental health.

More so when it comes to mental health, adolescents face challenges in obtaining relevant information. Lack of information and access to resources is associated with health and social ramifications, such as risky behavior, negative mental health, poor utilization of prevention services, poor diagnosis and lower treatment, lower compliance and adherence, and poor physical health (Kutcher et al., 2015). Therefore, direct and indirect efforts should be taken to lessen the severity of mental disorders, as they may negatively affect life achievements and educational outcomes. This makes schools a community resource for first-line support of prevention programs, because of the ability to reach larger populations of adolescents (Calear and Christensen, 2010). The optimal setting of schools makes teachers a distinct group, whose MHL is a key determining factor for students’ mental health. Primarily, teachers have multiple roles to play. They serve as a psychological knowledge base for information and raise awareness. Teachers may be responsible for the identification, early detection, and referral services for learners in need of psychological help. Teachers may serve as key players for information sharing to parents, family, children, and the social environment in their communities based on the four domains of transgenerational transmission of mental disorders (TTMDs) model outlined by Dobener et al. (2022). Teachers, by playing a role, may influence the prevalence of mental illness in communities, as they may address the five transmission mechanisms, namely, social factors, family, parent–child interactions, prenatal factors, and to a lesser extent genetic factors. However, lack of MHL has also been a key driver of some of the attitudes and beliefs attributed to mental illness, including stigmatization (Kapungwe et al., 2011). Therefore, teachers have an important function in managing learners’ mental health, educational needs, and social needs, and they are *ad hoc* gatekeepers to specialized services. A point to consider is that current school-based interventions are inadequate and fail to address learners’ mental health needs. Such discrepancies result in poor mental health support services and missed opportunities for risk children Brockman cited in Bruland et al. (2017). Befittingly, teachers’ attitudes and perceptions become an important factor in influencing and determining students’ mental health status and literacy (Chen et al., 2021).

Mental health literacy refers to knowledge, attitudes, beliefs, and skills related to mental health that emerge from experience, education, and existing belief systems (Jorm et al., 1997). MHL is a multidimensional phenomenon with the following six factors: identification of specific disorders and various forms of psychological distress; beliefs and information about risk factors and causes; information and understanding of self-help interventions; information and awareness about available services for professional help; mental health stigma; and information about how to seek help for mental health, including content related to improving positive mental health outcomes (Jorm, 2000). In addition to these constructs, scholars have adopted a definition of MHL as “knowledge, attitudes, and habits developed by individuals to promote the mental health of themselves and others and to cope with mental illnesses in themselves and others” (Jiang et al., 2020, p. 235). The iterative meaning of these definitions emphasizes fighting stigma, maintaining positive mental health, and empowering individuals to improve their help-seeking behaviors (Kutcher et al., 2015). Accordingly, higher levels of MHL, positive attitudes, beliefs, knowledge, and skills are critical for early recognition, prevalence, and incidence, as well as minimizing stigma around mental health disorders and illness (Jorm et al., 1997). Consistent with MHL constructs as explained, they direct the importance of teachers’ attitudes and perceptions in MHL. Teachers are important for promoting learners’ improvement of mental health at the micro-level, sharing information, and health-seeking behaviors, as well as informing policy frameworks at the macro-level (McKenzie, 2020).

In its assumption, this study adopts a qualitative case study design believing that teachers with high MHL will be associated with informed knowledge and recognition, thus improving health-seeking behaviors and reducing mental health stigma among learners and school authorities. This is supported by various studies, which posit that when teachers build strong positive relations with their learners, making them accountable for their work becomes more meaningful for learners suffering from depression and other mental health conditions (UCLA Center for Mental Health in Schools [UCLA], 2009). Therefore, teachers’ positive attitudes and perceptions play a critical role in learning, sharing knowledge on MHL, and improving learners’ wellbeing. However, despite the key role played by teachers in mental health, there exists a gap in knowledge that attests to the role of teachers in mental health; additionally, there is less information regarding teachers’ attitudes and perceptions of MHL in the context of adolescent mental health in schools. Bruland et al. (2017) confirms that the lack of MHL in teachers is notable when they fail to identify affected children, and in cases where they have been identified, teachers lack knowledge and face uncertainties in dealing with the mental health challenges of learners. Teachers’ attitudes and perceptions become a measure of success for appropriate interventions for learners with mental health challenges. This is because when teachers identify mental health challenges, teachers’ responses are not adequate and fulfilling for learners because of teachers’ attitudes, perceptions, and experiences, with mental health issues. Jorm (2012) points out that during the mid-1990s, mental health training focused on primary care mental health workers and general practitioners, and was not spread to communities like schools and teachers. Within the sub-Saharan region, the implementation of mental health services initiatives and intervention programs has been a challenge due to the lack of MHL (Kapungwe et al., 2011). The research study seeks to bridge the knowledge gap in perceptions and attitudes of teachers in MHL and adolescent learners’ wellbeing. The following questions were used to narrow the knowledge gap: (i) what knowledge do teachers have on MHL, (ii) what are the attitudes of teachers on MHL, (iii) what are the perceptions of teachers on MHL, and (iv) what measures can be used to improve teachers’ attitudes and perceptions on MHL?

## Materials and methods

A qualitative approach was chosen for this study to explore and understand the meaningsand interpretations of individuals and groups to the social challenges (Creswell, 2014). The meanings and interpretations studied were regarding mental health among adolescents, focusing on teachers’ attitudes and perceptions of MHL. A case of mental health challenge exists among adolescents, and it relates to everyday life experiences for learners and teachers in secondary schools. A case study allows for the exploration of real-life circumstances using multiple sources of information (teachers), allowing for case descriptions and themes within the study site (Odzi Secondary School) (Creswell, 2012). In the case study, design an array of purposive sampling is available for use to show different perspectives on the research being undertaken. More so, purposive sampling allows the researcher to use knowledge of a population that understands desired knowledge; therefore, participants were purposively sampled based on the judgment that participants will provide access to desired data, their willingness to be studied, and provide knowledge of the research topic (Creswell, 2012).

### Participants and study setting

A total of 12 teachers were purposively sampled from Odzi Secondary School in Mutare. The school is situated in the peripheral area of Mutare Urban. It is a subsistence farming area with heavy activities of illegal mining activities. The site was chosen purposively to inform an understanding of the research problem and the central phenomena in the study (Creswell, 2012). Purposively sampled participants had teaching experience of more than 4 years at the secondary level. The reason for selecting this sample was to have teachers who had observed learners from low secondary to middle-level secondary learners (form 1–4, usually aged 13–16 years). Five of the selected participants were teachers who had additional responsibilities in guidance and counseling, two were responsible for sports, and five were from general academia.

### Data collection tool

A semi-structured interview was developed for the study to be conducted one-on-one with participants. The framework of the interview protocol was crafted based on previous research studies on MHL and the research questions of the study. The overarching questions in the semi-structured interview protocol aim to understand the knowledge of teachers on MHL, attitudes of teachers on MHL, perceptions of teachers on MHL, and possible measures that can be used to improve teachers’ attitudes and perceptions of MHL. Semi-structured interviews provided participants with the opportunity to express views, opinions, and commentary on perceptions and attitudes on MHL.

### Data collection procedure

Participants who could best answer the questions were identified with the support of school administrators. One-on-one interviews were conducted with each of the participants. The interviews were conducted at the school premises where there were no distractions. Interviews were tape-recorded, and notes were taken for observational cues. Participants were allowed to respond to their choice of language Shona and English during the interview process. The duration of the interviews ranged from 45 min to 1 h. Interviews were transcribed in English for data analysis.

### Ethical considerations

Ethical approval was obtained from the Manicaland State University of Applied Sciences Research Board prior to visiting the study site and conducting semi-structured interviews. Permission to conduct semi-structured interviews at Odzi Secondary School was further granted by the school authorities. The briefing was conducted with all participants to explain the purpose and structure of the study. Detailed information was given to the participants in the information sheet where informed consent was signed for participating in the study and recording of interview(s). Participants were informed about their right to withdraw at any point of the study without any penalties and infringements on their rights. Participants were informed and guaranteed confidentiality and privacy during data reporting, analysis, and publication stages by coding them with an alphanumeric. Participants were informed that there was no potential harm either in form of physical or psychological nature; however, they were informed that they could not respond to some questions that may be of discomfort to their dispositions.

### Data analysis

Data were analyzed using Braun and Clarke’s (2006) thematic analysis. Repeated readings and coding by researchers were done to have a clear understanding and interpretations of meanings by participants and developed codes. Data codes were carefully synthesized during the iterative spiral nature of data analysis until a general consensus was reached. The analysis consisted of describing, classifying, and connecting data on the phenomena of teachers’ perceptions and attitudes on MHL (Byrne, 2022). Thematic analysis of gathered data allowed for synthesis, descriptions, and development of themes toward teachers’ perceptions and attitudes on MHL. Developed themes allowed for interpretation, meaning, and understanding of MHL by teachers, by defining concepts, mapping, creating typologies, finding associations, providing explanations, and developing strategies toward improving attitudes and perceptions of MHL for teachers (Byrne, 2022).

## Results

Emergent themes of the study were knowledge, lack of training and development, and stigma.

### Theme 1: Varying degrees of knowledge

Participants indicated varying degrees of knowledge about mental health and adolescent behavior. Knowledge was clustered around the domains like behavioral, psychological, and emotional wellbeing, as shown by excerpts below:

*Mental health is about individual behavior which is inconsistent to community social norms* (T2);

*Mental health is about an individual and psychological wellbeing* (T5);

*Mental health relates to individuals’ responses to environmental situations and their ability to adapt in these environments* (T8).

A general understanding of mental health existed among teachers as reflections showed variations in the understanding of mental health as a concept. However, there was a general limitation to the understanding of MHL as mostly emphasized assessment and diagnosis as a key construct.

*On mental health literacy I think it is about assessing learners if they are suffering from any mental health disorders* (T4);

*When we talk about mental health literacy, we are talking about diagnosing learners if they have any mental disorders* (T10).

Information on MHL was generalized. This was similarly noted in the general description and characteristics of mental disorders. The main discussed disorder was depression, anxiety, and disturbed thoughts.

*Most of our mental health challenges we face at school are depression, due to loss of a loved one* (T12);

*I have noted that sometimes some of the mental challenges are due to anxiety particularly during the exam period* (T10);

*Some of the learners particularly girls have anxiety and depression due to their bodily changes and it becomes worse when they get to their menstruation cycle* (T8);

*At school we sometimes get learners who are mentally disturbed as a result of* “*Ngozi,” some might have been intelligent but may start towards exams to behave abnormally (T4).*

### Theme 2: Teachers’ self-efficacy in dealing with mental health

There was a variation in the abilities of teachers to deal with mental health concerns among their learners.

*I have realised that when it comes to deal with girl issues on mental health is not my strongest and I cannot help, and usually look for a female teacher to address their concerns (T8);*

*From experience, I have found that workshops that I have attended on guidance and counselling have come in quite handy when giving counsel to students (T6);*

*Sometimes dealing with mental health issues is a challenge because of lack of information to the processes and steps to take to deal with the matter (T5).*

### Theme 3: Time limitations in addressing mental health at schools

Time was increased to have an influence on the attitudes and perceptions of teachers’ MHL, and the concern was competing for academic curriculum needs, which took more precedence over mental health education and practices.

*Putting in place instruction for guidance and counselling is very difficult because we be trying to catch up on academic content so there is limited time for concern in learner wellbeing (T5);*

*Our main focus when we get to school is to prepare learners for exams, COVID-19 as made things worse because we have to cover a lot of exam material and content (T8);*

*At the end of the day as teachers our assessment is based on the pass rate, and the pressure of work is over and we don’t put much time to mental health issues, even though we are aware of the importance (T11).*

### Theme 4: Stigma on mental health

Stigma on mental health issues was related to teachers’ attitudes on dating as well as witchcraft in the community.

*My concern is that some girls go out into relationships with some of these illegal miners and we cannot help them when they have stress (T6);*

*It’s difficult to deal with mental illness at school because of the belief in witchcraft in this community (T10);*

*At times we try to communicate with the parents sometimes it does not bear fruit and we leave the learners like that (T5);*

*You see some learners even how much we talk to them they seem not to change their behavior no matter how long counseling has taken place (T3).*

### Theme 5: Training and development in mental health

Training and capacity development was raised as intervention measure to address mental health issues at schools.

*It is important for us to be continuously trained and work in collaboration with other stakeholders in mental (T4);*

*For us to improve our support to students we need workshops that can help build capacity to deal with adolescent mental health issues at school, especially now, we have returned back to school after COVID-19 lockdowns (T6);*

*Workshops on mental health will help us improve on our strategies of improving learners’ mental health, especially when we are doing sports, we can use the opportunity to raise mental health awareness among our learners (T4);*

*As teachers we need to have the capacity to deal with mental health issues whether it’s a girl or boy, having this kind of information will help us to talk more about issues with learners and their parents as well (T3).*

## Discussion

The scope of MHL attitudes and perceptions in schools is driven toward the reduction and prevalence and mental health disorders in schools. Notably, “school is a suitable place to reach a high number of children and a setting where children are easily accessible for mental health promotion” (Bruland et al., 2017, p. 5).

Building and securing a strong foundation and knowledge for teachers is key in ensuring that school environment spaces are maximized for building positive mental health. In this scope, in-service training to build knowledge and capacity for teachers’ MHL may have an effect on adolescent mental health in schools (Kutcher et al., 2013). Wei et al. (2015) accord that designing programs for MHL for teachers can help change attitudes and perceptions on mental health and the reduction of stigma. This is, especially, important because adolescents who experience mental health challenges may have feelings of guilt, shame, fear of stigmatization, may be reluctant to ask for help or seek treatment, and are likely to isolate themselves with suicidal ideation/thoughts (Seven et al., 2021). Lack and limited knowledge have been mainly indicated as contributors to low self-efficacy and a poor approach to mental health by teachers (Walter et al., 2011). Therefore, increasing knowledge on mental health for teachers will increase self-efficacy in dealing with adolescent mental health, and acquired knowledge will boost their confidence levels in MHL. Based on the results, it can be noted that teachers face challenges in training and development; there is a high prevalence of mental health illness and challenges among learners based on the TTMD model and its transmission mechanisms in schools, but adequate training, sensitization, and awareness are not given to teachers to support learners that face mental health challenges (Bruland et al., 2017). The study, in particular, shows that teachers are widely untrained in identifying mental health issues (Schulze et al., 2019). With adequate training and sensitization, teachers can impart positive mental health outcomes. Improved and tailor-made capacity building for teachers may help in framing positive attitudes, and perceptions which may improve the uptake of self-help behaviors by learners, early detection, response, and reaction to mental health challenges in both the individual and those in their social network (Jorm, 2012). Training and development should occur in components and should include professional development, training of school health teams, and provision of educational resources; this includes curriculum support materials that align with educational outcomes to enable teachers to improve learners’ MHL (Milin et al., 2016; Cairns and Rossetto, 2019). Curriculum support will help teachers in addressing the challenges faced by time limitations. The instructional learning will make a shift from a strong orientation to academic focus on pass rate to the holistic development of a learner’s emotional and social competencies. Making health education a compulsory aspect of the curriculum may reduce stigmatization and improve health-seeking behaviors (Zimmerman et al., 2015). Additionally, time constraints are embedded by teachers finding a balance between playing the role of a teacher teaching a large class and attending to their academic social, and emotional needs. It entails finding a balance in satisfying their learners’ needs, as well as personal family and professional life, and ensuring a balance in the relationship based on their understanding of their limitations on their own influence puts an imbalance and constraining measure in teachers (Schulze et al., 2019). Having teachers well versed in depression literacy is crucial for the successful implementation of school-based programs. Optimization of depression literacy in students will have a major impact on student acquisition of the tools necessary to recognize depression and seek help should they need it (Miller et al., 2019). It is through education and experience of learning about mental disorders that professionals could become aware of the subtle and unintended ways in which pre-existing beliefs may be contributing to stigmatized attitudes (Knaak et al., 2017). Enacting policy measures that put emphasis on MHL in in-service teachers can help improve the capacity of teachers to deal with mental health in schools (Franklin et al., 2012; Kutcher and Wei, 2014). Therefore, introducing programs that deal with mental disorders among teachers can reduce negative perceptions and improve health-seeking behaviors for adolescents. Such programs should be directed by policy to be specific, developmentally appropriate, and effectively integrate into the existing social and organizational structures, such as school curriculums (Kutcher et al., 2016). Integrating MHL programs for teachers in schools can improve health literacy for adolescent learners; therefore, the incorporation may overcome misconceptions and stereotypes about mental health disorders in schools (Bröder et al., 2017).

## Conclusion

General interpretation and understanding in the present study showed that teachers who had undergone previous training had higher levels of knowledge toward MHL, among those who had not undergone previous training in mental health or guidance and couselling had low levels of knowledge in mental health. However, knowledge of these aspects was not put to use. The indication was an acknowledgment by teachers regarding their failure to notice mental health challenges among students. In some instances where mental health challenges were observed, teachers failed to offer or link students with appropriate support systems. It is imperative to increase MHL among teachers to increase their self-efficacy to deal with mental health issues among students. Building knowledge and capacity for MHL among teachers may help in transferring knowledge, raising mental health awareness, and early detection of mental health disorders among students. Effective development of curriculum for teacher development in MHL may provide favorable opportunities for improving MHL in schools, and these may achieve positive results with strong support in multi-sectoral and stakeholders’ interactive mechanisms. High levels of MHL among teachers have the potential of increasing confidence in making an informed strategic decision on the matter of adolescent mental health in schools. Such assertive measures could bring positive results for adolescents’ health-seeking behaviors and tendencies which can improve academic success and quality of life. It is critical that teachers and other school personnel are trained to recognize the red flags for potential mental health conditions.

## Limitations and future research

The limitations of the study are that the participants are drawn from a single peri-urban secondary school. There may be a need to cautiously guard against the generalization of results to other populations. Further studies may need to be carried out in different schools in Zimbabwe. The number of participants in this study 12 is limited. There is a need for further research with wide-ranging participants from different categories of schools in Zimbabwe and the Sub-Saharan region. The study was mainly focused on understanding the attitudes and perceptions of teachers towrd MHL in low-resourced schools, and more research in the form of randomized control trials is required, where interventions to mental health are conducted and the learners’ attitudes and understanding of mental health in low resource setting are studied.

## Data availability statement

The raw data supporting the conclusions of this article will be made available by the authors, without undue reservation.

## Ethics statement

The studies involving human participants were reviewed and approved by the Manicaland State University of Applied Sciences. The patients/participants provided their written informed consent to participate in this study.

## Author contributions

TS prepared the manuscript and data collection. IS collected and analyzed the data and reviewed the manuscript. MM collected the data and reviewed the manuscript. MK collected the data and analyzed and reviewed the manuscript. SS contributed to the design of the work, data analysis, and review of the manuscript. All authors contributed to the article and approved the submitted version.
